# Efficacy of Two-Stage Treatment of Onychomycosis Using a Long-Pulsed Nd:YAG 1064-nm Laser

**DOI:** 10.1155/2019/3647519

**Published:** 2019-06-16

**Authors:** Shan Zhong, Guan-ting Lin, Jun-ying Zhao

**Affiliations:** ^1^Department of Dermatology, Beijing Friendship Hospital, Capital Medical University, Beijing 100050, China; ^2^Department of Dermatology, Longyan First Hospital, Fujian Medical University, Longyan, Fujian 364000, China

## Abstract

**Objectives:**

Onychomycosis is a fungal infection of the nail bed, nail matrix, and nail plate. Lasers have recently been studied as future clinical options for onychomycosis. We sought to evaluate the efficacy of the long-pulsed Nd:YAG 1064-nm laser on onychomycosis using a two-stage treatment.

**Methods:**

A total of 100 affected nails (88 toenails and 12 fingernails from 22 patients) were treated using a long-pulsed Nd:YAG 1064-nm laser. The self-controlled treatment schedule included the first stage (1 session per week for 8 weeks) and the second stage (1 session every 4 weeks for 16 weeks). Patients were followed up with for 12 weeks after the last laser treatment. Direct microscopy, cultures, and clinical assessments were performed at various time points.

**Results:**

Of the nails studied, 84% were infected by* Trichophyton rubrum*, while others were coinfected with* T. rubrum* and other fugal strains. The mycological clearance rate and the clinical efficacy rate of the nails were 29% and 21% after the first stage, 69% and 35% after the second stage, and 67% and 39% during follow-up, respectively. The second stage of laser treatment significantly improved the mycological clearance and clinical efficacy.

**Conclusion:**

Long-pulsed Nd:YAG 1064-nm laser two-stage treatment was effective for onychomycosis, with significantly improved mycological clearance and clinical efficacy. This trial is registered with ChiCTR 1900021669.

## 1. Introduction

Onychomycosis is a chronic fungal infection of the nail plate, nail matrix, and nail bed that is more common in the toenails. The prevalence of onychomycosis is 2–13% [1], and up to 14–28% among elderly patients over 60 years old [2]. Onychomycosis can cause pain, discomfort, and cosmetic concerns and thus significantly impacts patients, both physically and emotionally [3]. Although there have been advances in current treatment options, including terbinafine and itraconazole, that have improved the complete cure rate to 70.5% [2], recurrence (relapse or reinfection) still occurs in 10–53% of patients [4]. Oral antifungal medications are associated with side effects, such as gastrointestinal upset, headaches, and liver dysfunction. Recently, laser treatments, including the use of the Nd:YAG 1064-nm laser, have shown encouraging and promising results in patients with onychomycosis [5–7]; however, there is no standard treatment course for laser treatment. Here, we report that a two-staged long-pulsed Nd:YAG 1064-nm laser treatment significantly improves onychomycosis.

## 2. Methods

### 2.1. Subjects

For this study, 22 patients (18 to 76 years old) with clinically and mycologically (both direct microscopy and cultures) diagnosed onychomycosis for at least one year were enrolled in the Department of Dermatology at Beijing Friendship Hospital. The Scoring Clinical Index of Onychomycosis (SCIO) [8] was 6–15 (grade II–IV). The study was approved by the Hospital Ethic Committee, and all patients signed written informed consent statements before the study started. Exclusion criteria included application of topical antifungal agents within the last months; application of systemic antifungal agents within the last 6 months; other nail diseases, such as lichen planus, psoriasis, atopic dermatitis, subungual hematoma, nevoid formation, or bacterial nail infections; pregnancy or lactation; and photo-hypersensitivity.

### 2.2. Treatment Procedure

**Figure 1:**
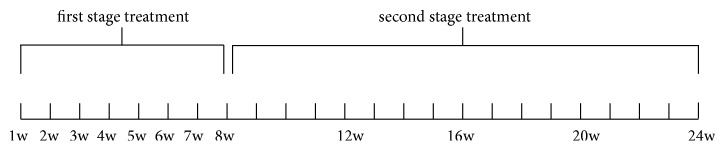
Two-stage treatment procedure. The entire procedure lasted 24 weeks.

Patients were treated with a long-pulsed Nd:YAG 1064-nm laser (Dualis SP; Fotona, Slovenia). The device fluency was set at 35–40 J/cm^2^, with a pulse duration of 35 ms, spot size of 4 mm, and frequency of 1.0 Hz. The fluency was chosen based on nail thickness, since thicker nails would require higher fluency. Each infected nail was treated with laser beam irradiation on the full nail plate and nail fold, using a spiral pattern that was repeated for 3 passes, with 2-min pause between passes. The two-stage laser treatment design for our study (Figure 1) included the first stage, which was performed once a week for eight weeks, and the second stage, which occurred once every four weeks for four visits. No local anesthesia or cooling device was utilized.

### 2.3. Treatment Evaluation

Direct microscopy, culture and clinical assessment were performed at 4, 8, 12, 16, 20, 24, and 36 weeks. The mycological efficacy was evaluated by the rate of simultaneous negative direct microscopy and negative cultures. Clinical efficacy was divided into four categories: cure (complete new nail growth with a smooth and brightly colored nail plate and less than 5% defects), significant improvement (≥ 60% new nail growth), improvement (≥ 20 and < 60% new nail growth), and inefficiency (< 20% new nail growth). The clinical efficacy rate is equal to the cure rate plus the significant improvement rate [7].

Patients were asked to report all adverse events directly during each laser treatment. A five-point Verbal Rating Scale (VRS; 0 = no pain, 1 = mild pain, 2 = moderate pain, 3 = severe pain, and 4 = intolerable pain) was used to evaluate pain. Patients were asked to mark the word (one of five that were given) that best fit the pain intensity during the laser treatment.

### 2.4. Statistic Analysis

An *χ*^2^ test was performed to determine the laser efficacy in all nails and to compare the SCIO grades and the degree of hyperkeratosis. P-values of less than 0.05 were considered statistically significance.

## 3. Results

### 3.1. Patient Characteristics

A total of 100 affected nails (88 toenails and 12 fingernails) from 22 patients (8 males and 14 females) were included, of which 37% were the nail of the big toe. The average age was 49.6 years (range from 27 to 76 years), and only three patients were older than 60 years. These patients exhibited all four clinical types of onychomycosis: distal and lateral subungual onychomycosis (DLSO), superficial white onychomycosis (SWO), proximal subungual onychomycosis (PSO), and total dystrophic onychomycosis (TDO) (Table 1). Overall, 84% of nails were infected by* T. rubrum*, 8% were coinfected with* T. rubrum* and* Candida albicans*, 4% were coinfected with* T. rubrum* and* Rhodotorula mucilaginosa*, and 4% were coinfected with* T. rubrum* and* C. parapsilosis*.

### 3.2. Mycological Efficacy

**Figure 2:**
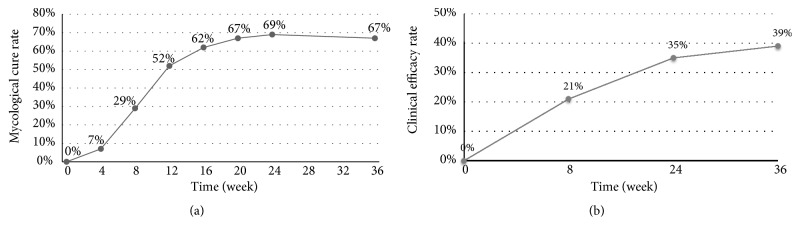
(a) Mycological cure rate and (b) clinical efficacy rate of nails that received laser treatment. There was a significant improvement in both rates at week 24, compared to week 8.

**Figure 3:**
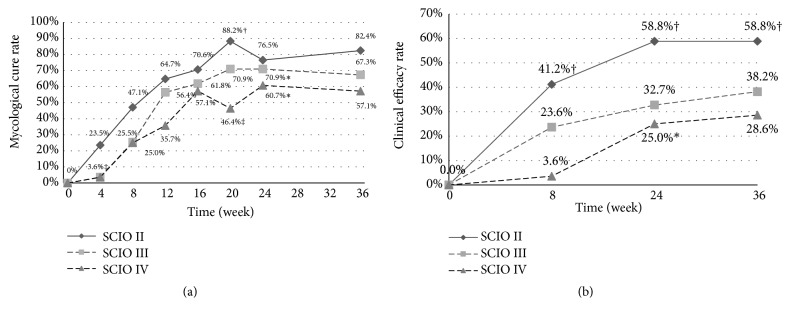
(a) Mycological cure rate of SCIO II, SCIO III, and SCIO IV during laser treatment. *∗p* < 0.05, comparing week 24 to week 8. ^†^*p* < 0.05, comparing week 20 to week 8. ^‡^*p* < 0.05, comparing SCIO IV to SCIO II at both week 4 and week 20. (b) Clinical efficacy rate of SCIO II, SCIO III, and SCIO IV during laser treatment. *∗p* < 0.05, comparing week 24 to week 8. ^†^*p* < 0.05, comparing SCIO IV to SCIO II at week 8, week 24, and week 36.

Two-stage laser treatment significantly improved the mycological cure rate (negative direct microscopy and negative culture, Figure 2(a)). Week 24 had a significantly higher number of mycological cures than week 8, suggesting the second stage is essential. Regarding each SCIO grade (Figure 3(a)), both SCIO III and SCIO IV were significantly improved at week 24 compared to week 8, and SCIO II was significantly improved at week 20 compared to week 8. The efficacy of the laser treatment was correlated with SCIO grade, in that the higher the SCIO grade, the lower the laser efficacy. Compared to the efficacy of SCIO IV, SCIO II had a significantly better efficacy at both week 4 and week 20.

### 3.3. Clinical Efficacy

The clinical efficacy significantly improved after two-stage laser treatment (Figure 2(b)). Week 24 had significantly higher clinical efficacy rates than week 8. Further, SCIO IV cases significantly improved at week 24 compared to week 8, and the clinical efficacy of SCIO II was significantly better than for SCIO IV at week 8, week 24, and week 36 (Figure 3(b)).

### 3.4. Pain and Side Effects

All patients reported various intensities of pain using the VRS. Most patients reported moderate pain, while none reported intolerable pain. All patients with mild pain (3 out of 3) were mycologically cured at week 36, whereas 8 out of 12 with moderate pain and 4 out of 7 with severe pain experienced mycological cures (Table 2). Treatment was tolerated by all subjects, and no side effects were reported at any time during the procedure.

### 3.5. Treatment Efficacy and Degree of Hyperkeratosis

**Figure 4:**
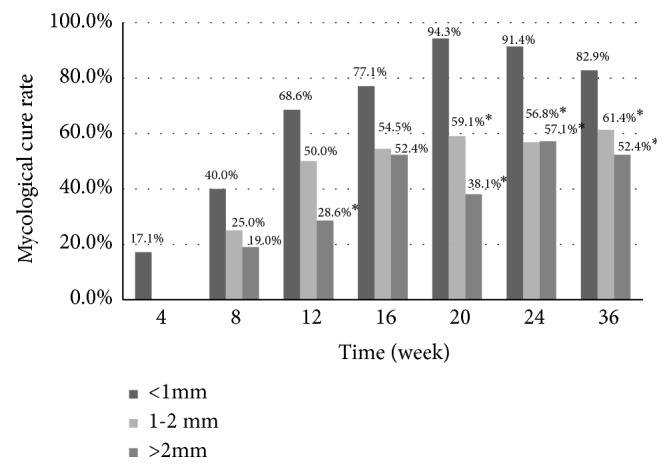
Mycological cure rate of hyperkeratosis from weeks 4 to week 36. The overall outcomes of hyperkeratosis of less than 1 mm exhibited significant (*∗p* < 0.05), which exhibited better effects than those with hyperkeratosis of 1–2 mm and more than 2 mm at week 20, week 24, and week 36.

The mycological cure rate was characterized by the degree of hyperkeratosis. Nails with fungal eradication and hyperkeratosis of less than 1 mm displayed the best outcomes after laser treatment, with effects starting to appear at week 4, whereas those with hyperkeratosis of 1–2 mm or greater than 2 mm saw effects around week 8. The overall outcomes for nails with hyperkeratosis of less than 1 mm exhibited significantly better effects than those with hyperkeratosis of 1–2 mm or greater than 2 mm at week 20, week 24, and week 36 (Figure 4).

### 3.6. Cosmetic Improvement after Laser Treatment and Nail Regrowth

**Figure 5:**
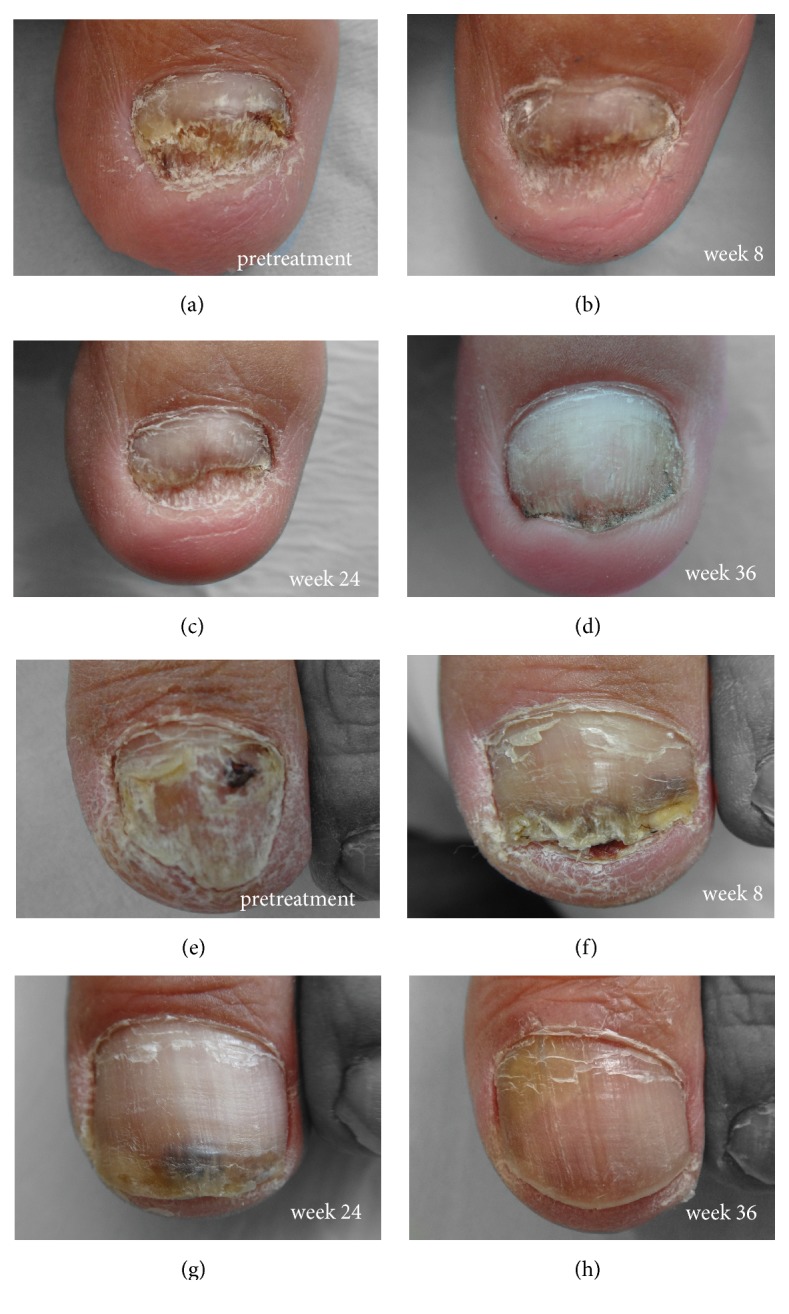
Representative photographs of the nails before and after laser treatment from two patients. Patient 1: (a) pretreatment, (b) week 8, (c) week 24, and (d) week 36. Patient 2: (e) pretreatment, (f) week 8, (g) week 24, and (h) week 36.

Nail appearances was also evaluated during the procedure. Laser sessions significantly improved cosmetic appearance at week 24 and week 36 (Figure 5), with similar effects being observed in most of the patients.

## 4. Discussion

The clinical treatment of onychomycosis remains extremely challenging due to the high recurrence rate. In general, current clinical interventions include oral, topical, and surgical therapies [2, 5]. Topical antifungal agents are not efficient in most cases because of the limited penetration of the nail plates. Systemic antifungal agents are considered the standard treatment, but long-term therapy, drug interactions, and side effects often restrain its applications [5–7].

Recently, the Nd:YAG 1064-nm laser was reported to inhibit the* in vitro* growth of* T. rubrum* [5, 9, 10]. Laser treatment disrupts the mitochondrial membrane potential by producing reactive oxygen species and inducing apoptosis in fungi [11]. Kozarev et al. [5] treated 194 nails from 72 onychomycosis patients with a long-pulsed (35 ms) Nd:YAG 1064-nm laser (Fotona, 35–40 J/cm^2^, spot size 4 mm) every week for 4 weeks, and the effects were encouraging. During the 3-month follow-up, 95.83% of patients had negative cultures. It is worth mentioning that the thicker nails were treated with 40% urea in this study. Hochman [12] reported negative cultures in 87.5% of 8 patients treated with a 0.65-ms pulsed Nd:YAG 1064-nm laser (LightPod Neo) using a fluency of 233 J/cm^2^ and 2-mm spot size for 2–3 treatments that occurred at least every 3 weeks. An antifungal cream was applied after each treatment.

We designed the long-pulsed Nd:YAG 1064-nm laser (Fotona) with the following parameters: 35–40 J/cm^2^, a pulse duration of 35 ms, a spot size of 4 mm, and a frequency 1.0 Hz. The mycological cure rate and the clinical efficacy rate of the nails were 29% and 21% at week 8 (first stage of treatment), 69% and 35% at week 24 (second stage of treatment), and 67% and 39% at week 36, respectively. The peak mycological efficacy rate occurred at week 24 and was stable until week 36, suggesting the laser therapy was effective. The peak clinical efficacy rate also occurred at week 24 and continued to increase through week 36. Long-term follow-up will be needed to determine the clinical recurrence time after laser treatment. Compared to other studies, the severity of onychomycosis, treatment course, treatment interval, parameters of the laser, evaluation criteria, and follow-up time varied, which may contribute to the efficacy of laser treatment. Therefore, randomized, intense follow-up clinical studies, including standard treatments and standard evaluation methods, will be conducted in the future to fully elucidate the efficacy of laser therapy on onychomycosis.

Our study demonstrated that efficacy significantly improved after the second stage of treatment compared to the first stage, suggesting that the second phase was necessary. Compared to other studies with only 8 sessions of laser treatment (one-stage), our study with 12 sessions laser treatment (two-stage) had better mycological efficacy rates. Lu [13] reported both clinical and mycological efficacy rates of 41.3% at week 36. Zhang [7] reported a clinical efficacy rate of 51%, with 13% positive fungal microscopy (100% at baseline) and 27% positive fungal culture (47% at baseline) at week 24. In Min Seok Kim's [14] study, the positive fungal microscopy rate at week 36 was 41.7% and 42.9% for groups A and B, respectively, and the positive fungal culture rate was 42.9% and 40%.

The efficacy of laser treatment also correlated with SCIO grade. Efficacy trend lines revealed that SCIO II was better than SCIO III, that SCIO III was better than SCIO IV, and that there was a significant difference between SCIO II and SCIO IV. Sergeev et al. [8] first published the SCIO to evaluate the severity of onychomycosis. Our results indicate that the SCIO may be used to provide laser treatment strategies: SCIO II and SCIO III patients should receive laser therapy alone (our team found the efficacy of systemic antifungals was equal to laser therapy in those patients [15]); SCIO IV patients should receive combination therapy (systemic antifungals, laser therapy, and topical therapy); and SCIO IV patients need a longer laser treatment course than SCIO II and SCIO III patients.

Regarding the degree of hyperkeratosis, we found that nails with hyperkeratosis of less than 1 mm had better outcomes. If nails thicker than 1 mm are treated with 40% urea before laser treatment, it could improve the overall efficacy.

In total, 84 nails from 19 patients were infected by* T. rubrum*, and 16 nails of 3 patients were coinfected with* T. rubrum* and other fugal strains. The mycological cure rate and clinical efficacy rate of nails with* T. rubrum* infection were 35% and 25% at week 8 (first stage of treatment), 69% and 38% at week 24 (second stage of treatment), and 68% and 45% at week 36, respectively. The mycological cure rate and clinical efficacy rate of nails with mixed fungal infections were 0% and 0% at week 8, 69% and 6.25% at week 24, and 63% and 6.25% at week 36, respectively. The mycological and clinical efficacy of laser treatment for nails with mixed fungal infections was much lower than the nails with* T. rubrum* infection alone.* T. rubrum* seemed to be more sensitive to laser intervention, possibly due to it containing more pigment than other fungi, such as* C. albicans* [16].

All patients experienced mild to severe pain during laser treatment, and thus, patients with high pain tolerance had better laser treatment efficacy. Laser therapy uses heat energy, which is the reason why the pain tolerant patients saw better outcomes. The question of whether it is necessary to use cooling device or anesthesia before laser treatment to get better effects is difficult to answer. Cooling devices or anesthesia may help patients to withstand the pain associated with treatment, so as to improve the laser effect, but there have been reports of nail loss as a result of overheating [11]. Further studies on the safety of laser therapy on the nail matrix need to be examined in depth.

## 5. Conclusions

In conclusion, two-stage long-pulsed Nd:YAG 1064-nm laser treatment exhibited an encouraging efficacy and safety modality in patients with onychomycosis.

## Figures and Tables

**Table 1:** Nail characteristics.

		N (%)
Clinical type	DLSO	87 (87%)
	SWO	3 (3%)
	PSO	2 (2%)
	TPO	2 (2%)
SCIO grade	II	17 (17%)
	III	55 (55%)
	IV	28 (28%)
Hyperkeratosis	< 1 mm	35 (35%)
	1–2 mm	44 (44%)
	> 2 mm	21 (21%)

DLSO: distal and lateral subungual onychomycosis, SWO: superficial white onychomycosis, PSO: proximal subungual onychomycosis, and TDO: total dystrophic onychomycosis.

**Table 2:** Pain measurement and number of mycologically cured cases at week 36.

Pain level (Verbal Rating Scale)	Number of patients	Number of mycological cures
no pain (0)	0	0
mild pain (1)	3	3 (100%)
moderate pain (2)	12	8 (66.7%)
severe pain (3)	7	4 (57.1%)
intolerable pain (4)	0	0

## Data Availability

The datasets generated and analyzed during this study are available from the corresponding author upon reasonable request.
